# A Surprising Decision

**Published:** 1892-10

**Authors:** 


### A SURPRISING DECISION.

SAN ANTONIO, Oct. 17.—Dr. James Kennedy sued a client for his fee for services rendered. Judge King to-day denied the doctor judgment on the ground that he had no certificate from the county board of medical examiners as the law required. There being no medical board in the county, it would appear that not only are all the physicians without legal recourse in collecting fees but are liable to indictment for practicing illegally.—*Daily Statesman.*

This is a very remarkable decision if it is correctly reported. The law on the subject of practicing medicine simply requires the possessor of a diploma—without discrimination as to kind of diploma or grade of standing of the medical school granting it—to have his diploma recorded in the office of the clerk of the county court in the county where he proposes to practice, a certificate of such registration or record being endorsed upon the margin of the diploma. Only those who are not graduates of a medical college, or others, who can not show a diploma (of some sort), are required by the law to appear before an examining board and obtain a license. Dr. Kennedy is a graduate of one of the best New York medical colleges; and it is not to be supposed that he ever thought of going before any board for examition and license.

The profession of Texas have fought against the law as it now stands on the statute books, and have insisted on a law requiring an examination, irrespective of diploma; but so far without avail. And so long as it is the law, we must abide by it; hence Judge King's decision is a great surprise to the profession; it virtually disfranchises every physician in Texas who is practicing by virtue of his diploma—even after he has complied with the registration requirement.

Dr. Kennedy will doubtless appeal from this unjust decision, and the result will be looked for with much interest, as it affects nearly every regularly educated physician in the State.

As poor as the law is with reference to examination of those who have no diploma, it is not enforced, because, we have always understood, there is a defect or flaw in the bill which renders it inoperative—the failure to define the offense—define what constitutes practicing medicine; and to appear before a board for examination and license is discretionary; there is no way to compel it; hence, when vacancies on boards have occurred the judges have not thought it worth while to fill them, and examining boards in most counties have ceased to exist, or, if existing, they do not pretend to meet, unless some applicant presents himself and asks for a license.

It looks to the JOURNAL like a remarkable construction Judge King has put upon the law. His construction is exactly in accordance with what *should be* the law. A diploma conveys no right or privilege, being, as defined by Webster, only "evidence of a degree having been conferred;" but by common consent it is, and for years has been, in nearly every State, accepted as authority unquestioned.

